# Supplementary material for: Does continuing learning help middle-aged and elderly people find employment in China? A propensity score matching analysis
Source: PLoS One. 2025 May 15;20(5):e0323743. doi: 10.1371/journal.pone.0323743 (PMC12080837; doi:10.1371/journal.pone.0323743)
Supplement: Attachment — Submitted filename: Response_to_Review_auresp_2.docx [file pone.0323743.s003.docx]

Dear Editor and Reviewers,

I would like to express my sincere gratitude for the opportunity to revise and resubmit our manuscript, "Does continuing learning help middle-aged and elderly people find employment in China? A propensity score matching analysis", for consideration for publication in PLOS ONE. The insightful comments and suggestions from the reviewers have greatly contributed to enhancing the quality and clarity of our research. We have carefully addressed each point raised during the review process and have made revisions as detailed below.

**Reviewer #2:**

1. The authors should cite the paper I recommend and highlight this paper's assumptions about fairness in the Discussion, which is more rigorous.

REPONSE: Thank you for your valuable comments. I have carefully considered your suggestion and have made the following revisions:

The two papers you recommended have been cited in the manuscript as references [24] and [34]. Additionally, I have highlighted the assumptions about fairness in the Discussion section, specifically in lines 240-242 and 304-308. I believe these revisions have enhanced the rigor of our manuscript.

2. The presentation of the paper needs further checking and standardisation.
For example, in line 128, there is a missing space before ‘(OLS)’.
Line 132, missing space before PSM.
A chinese character exists in Table I.
Formulas are missing punctuation and symbol descriptions.

REPONSE: Thank you very much for your detailed comments on the presentation of the paper. I have carefully reviewed the manuscript and made the following specific revisions to address the issues you pointed out:

Formatting and Consistency:

I have added the missing space before “(OLS)” in line 120.

I have added the missing space before “PSM” in line 124.

I have removed the Chinese character from Table I. This was likely a formatting error, and I have ensured that all tables are free from non-English characters.

Formulas and Punctuation:

I reviewed all formulas in the manuscript and ensured accuracy of formula expression and description

I have also conducted a thorough check of the entire manuscript to ensure consistency in formatting and presentation. I believe these revisions have significantly improved the overall quality and readability of the paper.
